# *In vitro* to clinical translation of combinatorial effects of doxorubicin and dexrazoxane in breast cancer: a mechanism-based pharmacokinetic/pharmacodynamic modeling approach

**DOI:** 10.3389/fphar.2023.1239141

**Published:** 2023-10-19

**Authors:** Hardik Mody, Tanaya R. Vaidya, Jovin Lezeau, Kareem Taha, Sihem Ait-Oudhia

**Affiliations:** ^1^ Center for Pharmacometrics and Systems Pharmacology, Department of Pharmaceutics, College of Pharmacy, University of Florida, Orlando, FL, United States; ^2^ Quantitative Pharmacology and Pharmacometrics (QP2), Merck & Co., Inc., Rahway, NJ, United States

**Keywords:** doxorubicin, dexrazoxane, breast cancer, pharmacodynamic drug-drug interaction, proof-of-concept study

## Abstract

Dexrazoxane (DEX) is the only drug clinically approved to treat Doxorubicin-induced cardiotoxicity (DIC), however its impact on the anticancer efficacy of DOX is not extensively studied. In this manuscript, a proof-of-concept *in vitro* study is carried out to quantitatively characterize the anticancer effects of DOX and DEX and determine their nature of drug-drug interactions in cancer cells by combining experimental data with modeling approaches. First, we determined the static concentration-response of DOX and DEX in breast cancer cell lines, JIMT-1 and MDA-MB-468. With a three-dimensional (3D) response surface analysis using a competitive interaction model, we characterized their interaction to be modestly synergistic in MDA-MB-468 or modestly antagonistic in JIMT-1 cells. Second, a cellular-level, pharmacodynamic (PD) model was developed to capture the time-course effects of the two drugs which determined additive and antagonistic interactions for DOX and DEX in MDA-MB-468 and JIMT-1, respectively. Finally, we performed *in vitro* to *in vivo* translation by utilizing DOX and DEX clinical dosing regimen that was previously identified to be maximally cardioprotective, to drive tumor cell PD models. The resulting simulations showed that a 10:1 DEX:DOX dose ratio over three cycles of Q3W regimen of DOX results in comparable efficacy based on MDA-MB-468 (additive effect) estimates and lower efficacy based on JIMT-1 (antagonistic effect) estimates for DOX + DEX combination as compared to DOX alone. Thus, our developed cell-based PD models can be used to simulate different scenarios and better design preclinical *in vivo* studies to further optimize DOX and DEX combinations.

## Introduction

Doxorubicin (DOX) is widely used to treat a variety of solid tumors and hematological malignancies in the clinic (Bonadonna et al., 1969; Thorn et al., 2011; Sritharan and Sivalingam, 2021). However, dose-limiting DOX-induced cardiotoxicity (DIC) hampers the extent of its clinical utility (Von Hoff et al., 1979; Swain et al., 2003). Depending on the underlying mechanisms involved, various strategies have already been explored to mitigate DIC (Wenning et al., 2019). Regardless of the cardioprotective strategy employed, only modest clinical improvement has been reported and the challenge to mitigate DIC in DOX-treated patients continues to exist. To date, the iron chelator Dexrazoxane (DEX) has been the only drug clinically approved to treat DIC (Seifert et al., 1994; Langer, 2014).

The cardioprotective activity of DEX has long been attributed to the iron-chelating activity of its metabolite, ADR-925 (Buss and Hasinoff, 1993; Hasinoff et al., 2003). This metabolite is involved in the sequestering of free ferrous and ferric ions that otherwise catalyze the formation of toxic hydroxyl radicals during the redox cycling of DOX in cardiomyocytes. It is also reported to competitively bind to the iron from the iron-DOX complex in the mitochondria, which otherwise contributes to cardiomyocyte stress and toxicity (Jirkovsky et al., 2018). Besides, DEX is also known to bind and inhibit the topoisomerase II enzymes (Lyu et al., 2007; Deng et al., 2014). While the binding and inhibition of topoisomerase IIβ contributes to the cardioprotective activity of DEX in cardiomyocytes, it can also potentially bind and inhibit its sister isoform, topoisomerase IIα which is highly expressed in cancer cells (Yan et al., 2009; Deng et al., 2014). Thus, through this mechanism, DEX can potentially interfere with the anti-cancer activity of DOX and may impact the intended DOX efficacy in the clinic.

While the cardioprotective effects of DEX have been well characterized in clinical and preclinical studies (Herman and Ferrans, 1990; Hochster et al., 1992; Speyer et al., 1992; Swain et al., 1997a; Swain et al., 1997b; Lebrecht et al., 2007; Popelova et al., 2009), the impact of DEX on the anticancer activity of DOX has not been studied extensively. As we further optimize and rationalize the combination regimen (e.g., doses, dosing interval, frequency, etc.) in the clinic, it is important to evaluate the combinatorial effects of DOX and DEX on the overall anticancer efficacy and strike a balance of safety and efficacy, i.e., to tap on the beneficial cardioprotective effects but avoid any unintended detrimental effects of DEX on efficacy. To address this gap, we previously carried out quantitative characterization of the cardioprotective effects of DEX on DIC in cardiomyocytes via a combination of experimental and computational approaches (Mody et al., 2023). Our findings based on the *in vitro*-*in vivo* predictions indicated that DEX and DOX dose ratio of 10:1 and the Q3W (once every 3 weeks) DOX regimen offer maximal cardio-protection.

Building upon previous learnings, a proof-of-concept study is further presented in this manuscript, with the aim now to quantitatively characterize the anticancer effects of DOX and DEX, as single agents as well as for the combination in cancer cell lines. Breast cancer was selected as the prototype oncological disease and two cancer cell lines previously reported to be sensitive to DOX, JIMT-1 and MDA-MB-468, were used in the study. These cell lines were also selected based on the two difficult-to-treat breast cancers they represent; breast cancer resistant to HER2 therapy for JIMT-1 cells and triple negative breast cancer for MDA-MB-468 (Tanner et al., 2004; Chavez et al., 2010). As with the previous study (Mody et al., 2023), a similar approach of combining experimental data with modeling and simulation (M&S) strategies was employed. First, the concentration-response curves for DOX and DEX as single agents in both the cancer cell lines were investigated across a range of physiologically relevant concentrations. Based on this, respective *in vitro* IC50s were determined and DOX and DEX concentrations were selected for subsequent drug combination and time-course studies. A three-dimensional (3D) response surface analysis using a competitive interaction model was conducted to determine the nature of their interactions. Subsequently, a mathematical, cellular response, pharmacodynamic (PD) model was developed to capture the time-course effects of the two drugs as single agents as well as their dynamic drug-drug interaction in cancer cells, and relevant parameters were estimated. Finally, *in vitro* to *in vivo* translation was performed by utilizing clinically relevant doses and regimens of DOX and DEX, previously identified to be maximally cardioprotective (Mody et al., 2023), to drive tumor cell PD models and to assess long-term effects of this combination on tumor cell killing. Thus, the established PK/PD models along with long-term simulations demonstrated their utility in testing clinical scenarios and can be further used to design subsequent preclinical *in vivo* studies aimed to better optimize DOX and DEX combinations to mitigate toxicity while retaining efficacy.

## Materials and methods

### Drugs, reagents, and cell line

Doxorubicin HCl (DOX) was purchased from Selleck Chemicals (Houston, TX) while Dexrazoxane (DEX) was from Millipore Sigma-Aldrich Co. (St. Louis, MO). DOX was dissolved in molecular biology grade water while DEX in DMSO as per manufacturer’s instructions. Stock solutions were stored at −80°C for long-term storage while fresh serial dilutions were prepared in cell culture media each time prior to experiments. JIMT-1 cells were procured from AddexBio (San Diego, CA) and cultured in cell culture media comprising of Dulbecco’s Modified Eagle’s Medium (DMEM) with 10% sterile filtered fetal bovine serum (FBS), 1% sodium bicarbonate, 1% MEM Non-essential amino acids, and 1% penicillin/streptomycin antibiotics. MDA-MB-468 cells were purchased from American Type Culture Collection (ATCC) (Manassas, VA) and cultured in DMEM with 10% FBS, and 1% penicillin/streptomycin. Both the cell lines were maintained at 37°C in a humidified atmosphere with 5% CO_2_ and passaged upon confluency with 0.25%Trypsin/2.21 nM EDTA. Details regarding the remaining reagents have been previously described elsewhere (Mody et al., 2023).

### CCK-8 cell viability assay

Based on growth patterns, JIMT-1 or MDA-MB-468 cells were seeded at a density of 3 × 10^3^ or 5 × 10^3^ cells per well (100 µL) of a 96-well plate. After overnight incubation, the cell lines were exposed to varying concentrations of DOX (0.005–1 µM), DEX (0.1–400 µM), or their combinations for 72–96 h for various sets of experiments. The CCK8 cell viability assay was subsequently carried out as per the manufacturer’s instructions and as described previously (Mody et al., 2023). More specifically, at the end of the treatment period, the treated or control cells were incubated with the CCK8 solution for an hour (10 µL/well for a 96-well plate). Subsequently, a microplate spectrophotometer (Biotek, Winooski, VT) was used to measure the absorbance at 450 nm. Experiments were performed in at least triplicates for each experimental condition and compared against appropriate vehicle controls.

### Mathematical modeling

#### Concentration-response relationships and determination of IC50

An inhibitory Hill function was used to characterize the concentration-response curves for DOX and DEX in the two breast cancer cell lines, JIMT-1 and MDA-MB-468 at 72 h and to estimate their corresponding maximal effects (*I*_*max*_) and concentrations required to achieve 50% of maximal effects (*IC*_*50*_).

$$R=R_{0}∙\left(1-\frac{I_{\max}∙C}{{IC}_{50}+C}\right)$$ (1)

where *R* is the response to drug treatments (% cell viability), *R*_*0*_ is the baseline response (% cell viability in absence of drug treatments or control), *I*_*max*_ is the maximal effect of drug treatments, *IC*_*50*_ is the drug concentration required to achieve 50% of *I*_*max*_, *C* is the drug concentration. The *IC*_*50*_ values were used to select concentrations for single agents and combinations during time-course and subsequent studies.

#### Evaluation of static concentration-response drug-drug interactions

Static joint effects of DOX and DEX on cell viability of JIMT-1 and MDA-MB-468 cells were evaluated by fitting a competitive interaction model (Chakraborty and Jusko, 2002; Pawaskar et al., 2013) to the concentration-response data for the combinatorial treatment as follows:

$$R=R_{0}.\;\left[1-\frac{\left(I_{\max,A}∙{\left(\frac{C_{A}}{\psi .IC50_{A}}\right)}^{\gamma_{A}}\right)+\left(I_{\max,B}∙{\left(\frac{C_{B}}{\psi .IC50_{B}}\right)}^{\gamma_{B}}\right)}{{\left(\frac{C_{A}}{\psi .IC50_{A}}\right)}^{\gamma_{A}}+{\left(\frac{C_{B}}{\psi .IC50_{B}}\right)}^{\gamma_{B}}+1}\right]$$ (2)

Where, R is % cell viability, R_0_ is % cell viability at baseline (i.e., 100%), I_max,A_ and I_max,B_ are the maximal effects of the two drugs (i.e., maximal fractions of inhibition), C_A_ and C_B_ are concentrations of the drugs, IC50_A_ and IC50_B_ are the half-maximal inhibitory concentrations of the drugs, γ_A_ and γ_B_ are the respective Hill coefficients and ψ is the interaction term. All individual drug-related parameters were fixed from the DOX and DEX concentration-response curve fittings and the interaction term, ψ, was estimated using Monolix software version 2016R1 (Antony, France: Lixoft SAS, 2016). The apparent interaction between the drugs was antagonistic when ψ > 1, synergistic when ψ <1 and additive when ψ = 1.

Additionally, three-dimensional (3D) response surface plots of cell viability versus DOX and DEX concentrations were constructed to visually evaluate the interaction between the drugs for both cell lines. Briefly, an additive interaction was assumed between both drugs (i.e., ψ = 1) and 3D response surface plots were generated for both cell lines using Eq. 2 with MATLAB version 2017a (The MathWorks, Inc., Natick, Massachusetts, United States). Next, observed cell viability data was overlaid on the additive response surface plots to assess location of the data points relative to the additive surface, thus enabling visual assessment of additive, antagonistic or synergistic effects between DOX and DEX.

#### Development of the cellular level pharmacodynamic (PD) model

The *in vitro* cellular level pharmacodynamic model (PD) was developed for the single agents, DOX, DEX, as well as their combination effects on the cell viability of human breast cancer cell lines, JIMT-1 and MDA-MB-468 as described below.

First, the degradation kinetics of DOX and DEX in cell culture media described previously (Jirkovsky et al., 2018; Vaidya et al., 2021) were leveraged. The equations describing their degradation kinetics are the same as used in the previous study (Eqs 1, 2 from (Mody et al., 2023)). Overall, the time dependent changes in the concentrations of DOX and DEX in the cell culture media were used to drive the cellular-level PD model described below. In addition, it was assumed that the two drugs do not interfere with each other’s degradation kinetics in the cell culture media for their combination group.

Next, the cell growth of breast cancer cell lines, JIMT-1 and MDA-MB-468, without any treatment (control group) was best described by an exponential growth function as follows:

$$\frac{dR}{dt}=k_{g}.R;R\;\left(0\right)=R_{0}$$ (3)

where *R* is the % cellular viability (cellular response) at time *t*, *k*_*g*_ is the first-order growth rate constant for cancer cell lines, while *R*_*0*_ is the % cellular viability at time zero.

##### Single agent PD models

The cellular response or the PD effect of DOX or DEX on the cell viability of JIMT-1 and MDA-MB-468 was described by a stimulatory effect (Hill function) on the cell death of cancer cells. A stimulatory effect on cell death in the JIMT-1 and MDA-MB-468 cancer cells lines was needed to capture the significant reduction in cell viability as compared to the control arm, as suggested by the observed data. The delay between the exposure of DOX or DEX and the non-linear cytotoxic (stimulation of death) effect on JIMT-1 and MDA-MB-468 was captured with the inclusion of three transit compartments for both drugs. This is consistent with previously published reports describing their mechanisms including temporal delay due to intracellular signaling cascade involved in their cytotoxic effects. It is to be noted that several empirical functions for cell-killing such as linear, power or sigmoidal (Hill) functions were initially evaluated to describe the trends in the observed data. Furthermore, the number of transit compartments were also varied for model optimization. The cell-killing function and number of transit compartments in the final models were selected based on visual inspection, goodness-of-fit and precision on parameter estimates. The differential equations for the effect of DOX or DEX as single agents on JIMT-1 and MDA-MB-468 cells in the final model are as follows:

$$K_{x}=\frac{S_{\max,x}∙C_{x}}{{SC}_{50,x}+C_{x}}$$ (3a)

$$\frac{{dK1}_{x}}{dt}=\frac{1}{\tau_{x}}.\left(K_{x}-{K1}_{x}\right);{K1}_{x}\left(0\right)=0$$ (3b)

$$\frac{{dK2}_{x}}{dt}=\frac{1}{\tau_{x}}\;.\left({K1}_{x}-{K2}_{x}\right);\;{K2}_{x}\;\left(0\right)=0$$ (3c)

$$\frac{{dK3}_{x}}{dt}=\frac{1}{\tau_{x}}\;.\left({K2}_{x}-{K3}_{x}\right);\;{K3}_{x}\;\left(0\right)=0$$ (3d)

$$\frac{dR}{dt}=k_{g}.R-{K3}_{x}\;.\;R;\;R\;\left(0\right)=R_{0}$$ (3e)

where the subscript x represents either DOX or DEX, *S*_*max,x*_ is the maximal killing rate constant while *SC*_*50,x*_ is the concentration of DOX or DEX required to induce half-maximal effect in JIMT-1 and MDA-MB-468. *K*_*x*_ is the cytotoxicity Hill function, *K1*_*x*_ to *K3*_*x*_ are transit compartments with *Ʈ*_*x*_ the mean transit time between compartments and C_*x*_ represents the drug concentration.

##### Drug combination PD models

As both the drugs induce cytotoxic effects (stimulation of death function) in JIMT-1 and MDA-MB-468, an interaction parameter, ψ, was applied to Eq. 4a (as shown below) (Zhu et al., 2015; Miao et al., 2016). If ψ = 1, then the interaction is additive, if ψ < 1 then synergistic, and if ψ > 1, then it is an antagonistic interaction. The differential equations for the combination effect of DOX and DEX in JIMT-1 and MDA-MB-468 are as follows:

$$K_{DOX}=\frac{S_{\max,DOX}∙C_{DOX}}{\left({SC}_{50,DOX}∙\psi \right)+C_{DOX}}$$ (4a)

$$\frac{{dK1}_{DOX}}{dt}=\frac{1}{\tau_{DOX}}∙\left(K_{DOX}-{K1}_{DOX}\right);{K1}_{DOX}\left(0\right)=0$$ (4b)

$$\frac{{dK2}_{DOX}}{dt}=\frac{1}{\tau_{DOX}}∙\left({K1}_{DOX}-{K2}_{DOX}\right);\;{K2}_{DOX}\left(0\right)=0$$ (4c)

$$\frac{{dK3}_{DOX}}{dt}=\frac{1}{\tau_{DOX}}∙\left({K2}_{DOX}-{K3}_{DOX}\right);\;{K3}_{DOX}\left(0\right)=0$$ (4d)

$$K_{DEX}=\frac{S_{\max,DEX}∙C_{DEX}}{{SC}_{50,DEX}+C_{DEX}}$$ (4e)

$$\frac{{dK1}_{DEX}}{dt}=\frac{1}{\tau_{DEX}}∙\left(K_{DEX}-{K1}_{DEX}\right);\;{K1}_{DEX}\left(0\right)=0$$ (4f)

$$\frac{{dK2}_{DEX}}{dt}=\frac{1}{\tau_{DEX}}∙\left({K1}_{DEX}-{K2}_{DEX}\right)\;;\;{K2}_{DEX}\;\left(0\right)=0$$ (4g)

$$\frac{{dK3}_{DEX}}{dt}=\frac{1}{\tau_{DEX}}∙\left({K2}_{DEX}-{K3}_{DEX}\right);\;{K3}_{DEX}\left(0\right)=0$$ (4h)

$$\frac{dR}{dt}=k_{g}∙R-\left({K3}_{DOX}+{K3}_{DEX}\right)∙R;\;R\;\left(0\right)=R_{0}$$ (4i)

#### *In Vitro–in vivo* translation of PD responses

*In vitro* to *in vivo* translation of PD responses for the joint effect of DOX + DEX in JIMT-1 and MDA-MB-468 cells was performed by utilizing clinically relevant pharmacokinetic (PK) profiles of the two agents to drive the PD models developed in the *in vitro* setting. In the previous study assessing cardioprotective activity of the DOX + DEX combination (Mody et al., 2023), it was determined that a DEX:DOX dose ratio of 10:1 or 20:1 is predicted to provide maximal cardioprotective effects. Furthermore, DOX dose-fractionation was not predicted to improve cardioprotective activity of the combination. Thus, a clinically relevant DOX dosing regimen of 50 mg/m^2^ administered every 3 weeks (Q3W) alone or in combination with DEX 500 mg/m^2^ over three cycles was chosen for performing model simulations for cell viability of JIMT-1 and MDA-MB-468 cells using the PD models established *in vitro*.

First, DOX and DEX plasma PK data was obtained from previously published reports (Earhart et al., 1982; Kontny et al., 2013) and characterized with PK compartmental models as described previously (Mody et al., 2023). Next, DOX tumor concentrations were determined by using tumor distribution data in humans as reported in He et al. (2018). Briefly, tumor tissue and plasma concentration-time data were digitized using WebPlotDigitizer Version 4.3 (Pacifica, CA, USA) and the ratio of area under the concentration-time curves was calculated as AUC_tumor_/AUC_plasma_ with the linear trapezoidal method using Phoenix WinNonlin (Version 8.2). Subsequently, this ratio was used as a multiplicative factor (Fac_1_) to determine DOX tumor concentrations as a fraction of the simulated plasma concentrations of DOX for the 50 mg/m^2^ Q3W regimen. Similarly, DEX tumor concentrations were determined using a multiplicative factor, Fac_2_, as a fraction of the simulated plasma concentrations of DEX at a dose level of 500 mg/m^2^. Due to unavailability of tumor distribution data for DEX, arbitrary values of Fac_2_ (0.1, 1 and 10) were used to simulate various scenarios for DEX distribution to tumor tissue in humans.

The determined tumor concentrations were then used to drive the *in vitro* PD models established for JIMT-1 and MDA-MB-468 cells. Population simulations were conducted for 500 subjects with an arbitrary inter-individual variability (IIV) of 10% introduced on the PD parameters and the Fac_1_ and Fac_2_ parameters for each cell model. Evaluation of efficacy was performed by calculating the area under the effect (% cell viability) curve (AUEC) as an integrated measure of PD response for the following scenarios: DOX alone, DOX + DEX (Fac_2_ = 0.1), DOX + DEX (Fac_2_ = 1) and DOX + DEX (Fac_2_ = 10).

All PK-PD model fittings and simulations were performed using Monolix suites version 2016R1 or higher, while AUEC calculations were performed using RStudio version 1.2.5033.

## Results

### Concentration-response curves and determination of IC50 for DOX and DEX

**FIGURE 1:**
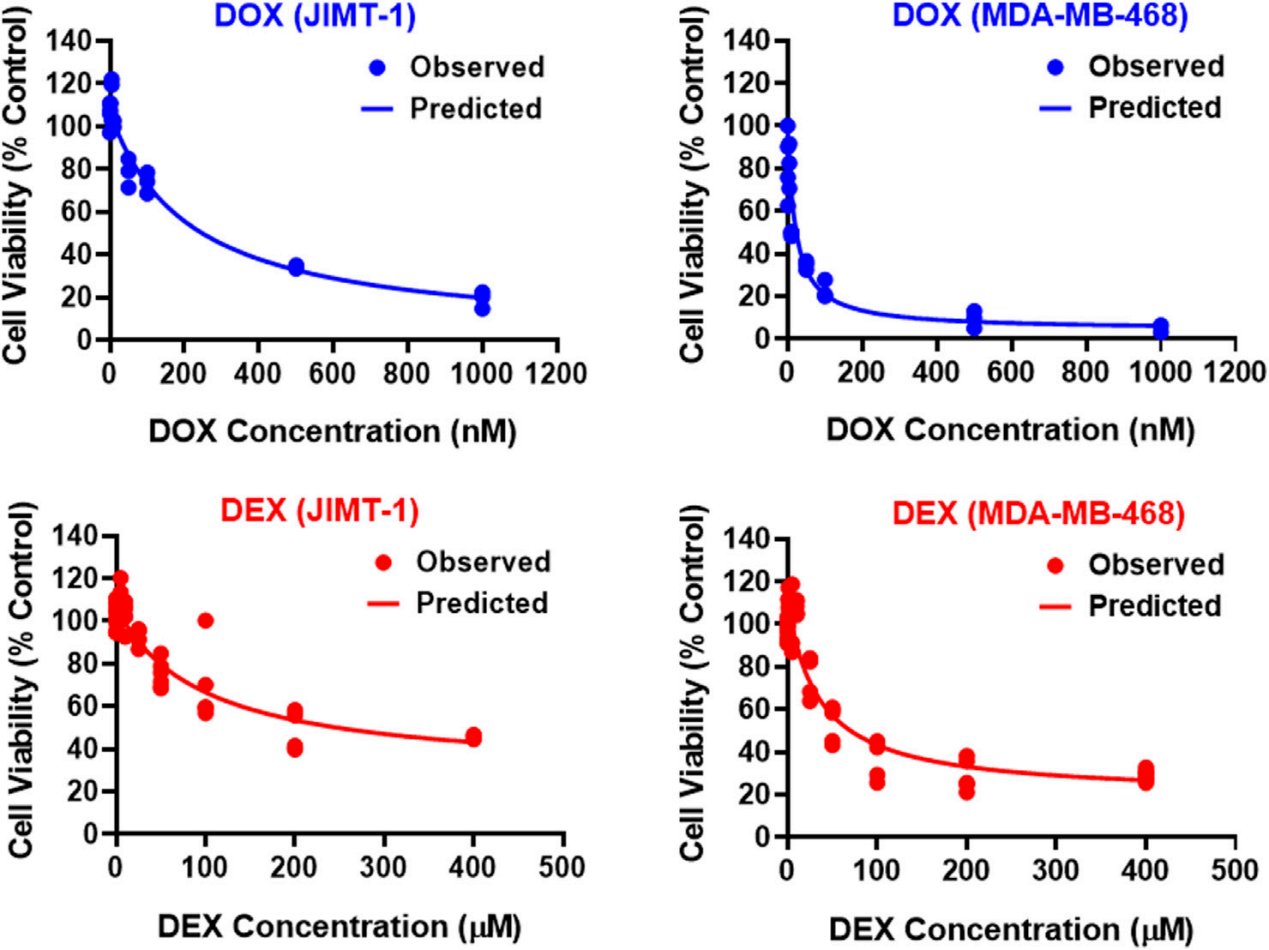
Concentration-response curves for DOX (top; blue) and DEX (bottom; red) as single agents in JIMT-1 (left) and MDA-MB-468 (right) cancer cell lines. All observed data are represented by solid circles while the smooth lines are model fittings.

The two breast cancer cell lines, JIMT-1 and MDA-MB-468, were exposed to a wide range of single agent (either DOX or DEX) concentrations for 72 h. The inhibitory Hill model was fitted to the concentration-response curves, each for DOX and DEX as single agents in both the cell lines as shown in Figure 1 and parameter estimates are summarized in Table 1. As expected, DOX was consistently more potent (∼500 to 2000 folds) as compared to DEX in both the cell lines. The IC50 for DOX was 214 nM and 21.2 nM while that of DEX was 97.5 µM and 36 µM in JIMT-1 and MDA-MB-468, respectively. In addition, DOX showed a higher maximal killing of cancer cell lines compared with DEX, as is evident by the estimated I_max_ of 0.988 and 0.959 (both close to 1) for the former compared with that of 0.74 and 0.825 for the latter in JIMT-1 and MDA-MB-468. Overall, MDA-MB-468 was more sensitive as compared to JIMT-1 for both DOX and DEX treatments as evidenced by a lower determined IC50 and a higher maximal killing for both DOX and DEX. The inhibitory Hill model captured the concentration-response data as demonstrated with observations versus individual prediction plots shown in Supplementary Figure S1.

**TABLE 1:** Concentration-response curve parameter estimates for DOX and DEX as single agents in JIMT-1 and MDA-MB-468 cancer cell lines.

Parameter (Units)	Definition	Estimate (% RSE)			
		DOX		DEX	
		JIMT-1	MDA-MB-468	JIMT-1	MDA-MB-468
R_0_ (%)	Baseline % cell viability	106	100	106	110
		(2.69)	(Fixed)	(2.35)	(3.62)
IC_50_ (nM or µM)	Drug concentration inducing 50 % of maximal effect	214 nM	21.1 nM	97.5 µM	36 µM
		(18.5)	(19.9)	(23.3)	(16.2)
I_max_	Maximal effect	0.988	0.959	0.74	0.825
		(3.7)	(1.27)	(6.77)	(2.17)

% RSE, % relative standard error in the model parameters.

### Static concentration-response combinatorial drug effects between DOX and DEX

**FIGURE 2:**
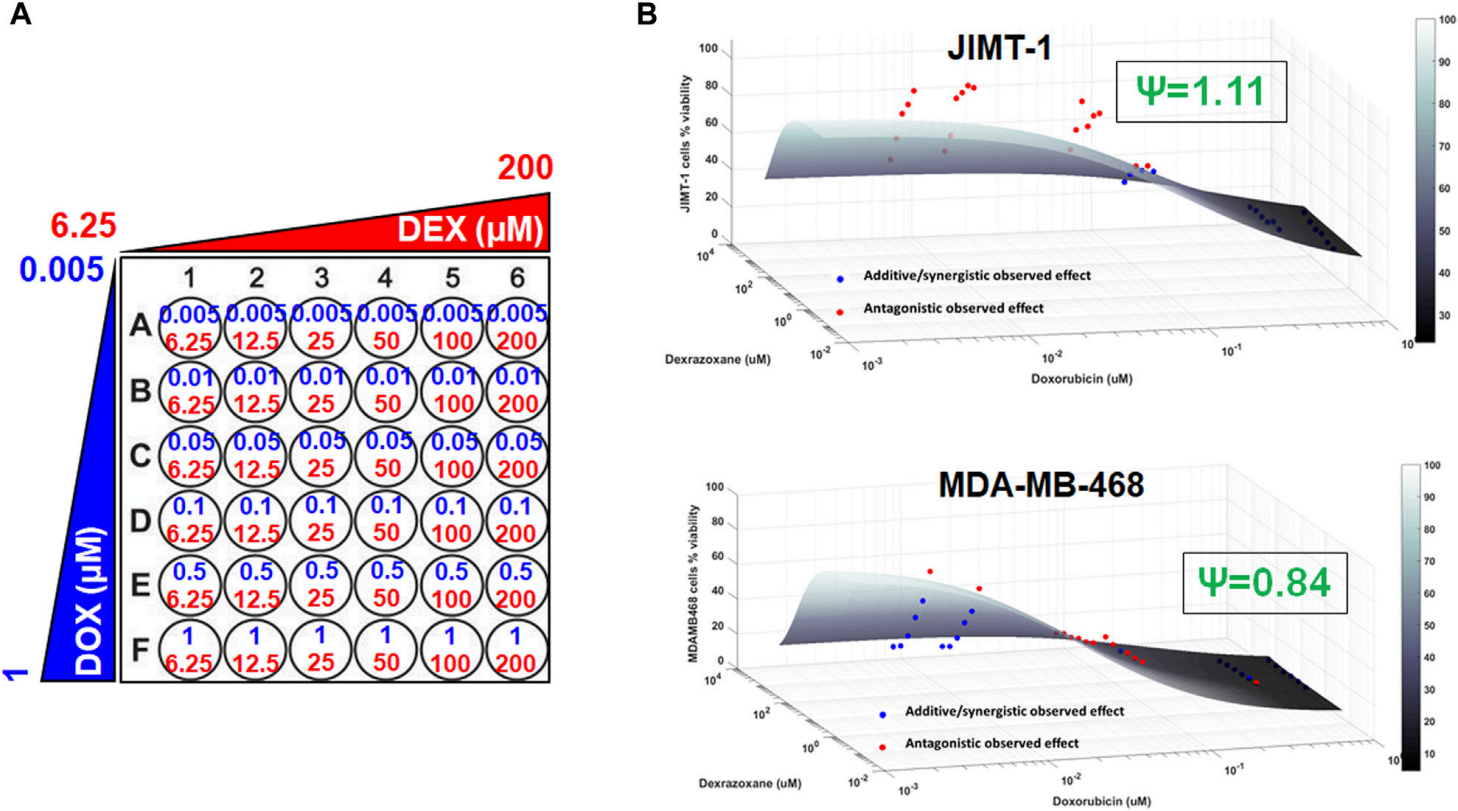
3D response surface plots using a competitive interaction model from the effects of single agent (DOX or DEX) and drug combination data in JIMT-1 and MDA-MB-468 cells. The concentrations used for single agents, DOX (0.005–1 µM) or DEX (6.25–200 µM) and their combinations are summarized in **(A)**. The 3D response surface plots represent model simulations under the assumption of an additive interaction (ψ = 1) and the circles represent observed cell viability data for JIMT-1 (**B**, top) and MDA-MB-468 (**B**, bottom), respectively. Circles above the surface are red in color and indicate antagonistic interactions while circles at or below the surface are blue in color and indicate additive or synergistic interactions between DOX and DEX. ψ = 1.11 and ψ = 0.84 indicate interaction parameters estimated using a competitive interaction model for JIMT-1 and MDA-MB-468 cells, respectively, and are consistent with the patterns of observed data relative to the additive 3D surfaces as described in the *Results* section.

To examine the cancer cell killing effects of the drug combinations and to determine the nature of their interactions in the static setting, cells were exposed to a range of DOX and DEX concentrations as single agents as well as their combinations for 72 h as indicated in Figure 2A. Six concentrations of DOX (from 0.005 µM to 1 µM), 6 concentrations of DEX (from 6.25 µM to 200 µM) and 36 different DOX and DEX combinations were used in both cell lines. A competitive interaction model was fitted to the above data (Chakraborty and Jusko, 2002; Pawaskar et al., 2013). In addition, 3D response surfaces were plotted under the assumption of additive interaction (ψ = 1) and observed data were overlaid on the plots (Figure 2B). Based on this analysis, the interaction parameter, ψ, was estimated at 1.11 for JIMT-1 and 0.84 for MDA-MB-468 cells. Thus, the drug combinations were determined to be modestly antagonistic (ψ slightly greater than 1) and modestly synergistic (ψ slightly less than 1) for JIMT-1 and MDA-MB-468, respectively. This was also reflected in the 3D plots (Figure 2B), wherein, a majority of the observed data points lay above the additive response surface for JIMT-1 cells, especially at the lower concentrations of DOX, indicating a mildly antagonistic effect. For MDA-MB-468 cells, several observed data points were below the additive surface and some on the surface indicating mild synergism between DOX and DEX.

### Time course effects of DOX and DEX as single agents and in combination

**FIGURE 3:**
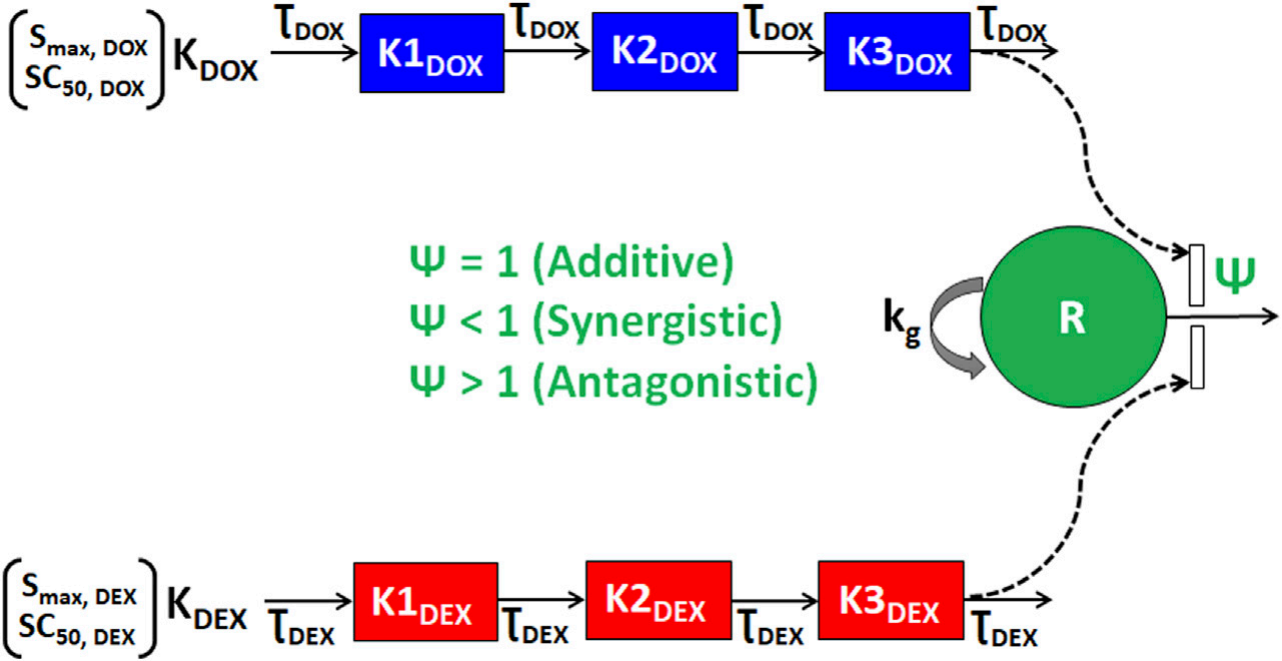
Schematic of the *in vitro* cellular-level pharmacodynamic model (PD) for the single and combinatorial effects of Doxorubicin (DOX) and Dexrazoxane (DEX) on human breast cancer cell lines (JIMT-1 and MDA-MB-468). Definitions of parameters for the model are listed in Table 2. The solid lines with arrows denote turnover of the indicated response. The green circle represents cell viability, the blue or red boxes represent transit compartments to describe the delay in the effects of DOX or DEX, respectively and the open solid rectangles represent the stimulation of death induced by DOX and/or DEX as single agents or combination and as indicated by dashed black arrows. The interactions of DOX and DEX on the stimulation of death of breast cancer cells is captured by ψ with ψ = 1, ψ < 1, or ψ > 1 indicating additive, synergistic, or antagonistic interactions, respectively.

**FIGURE 4:**
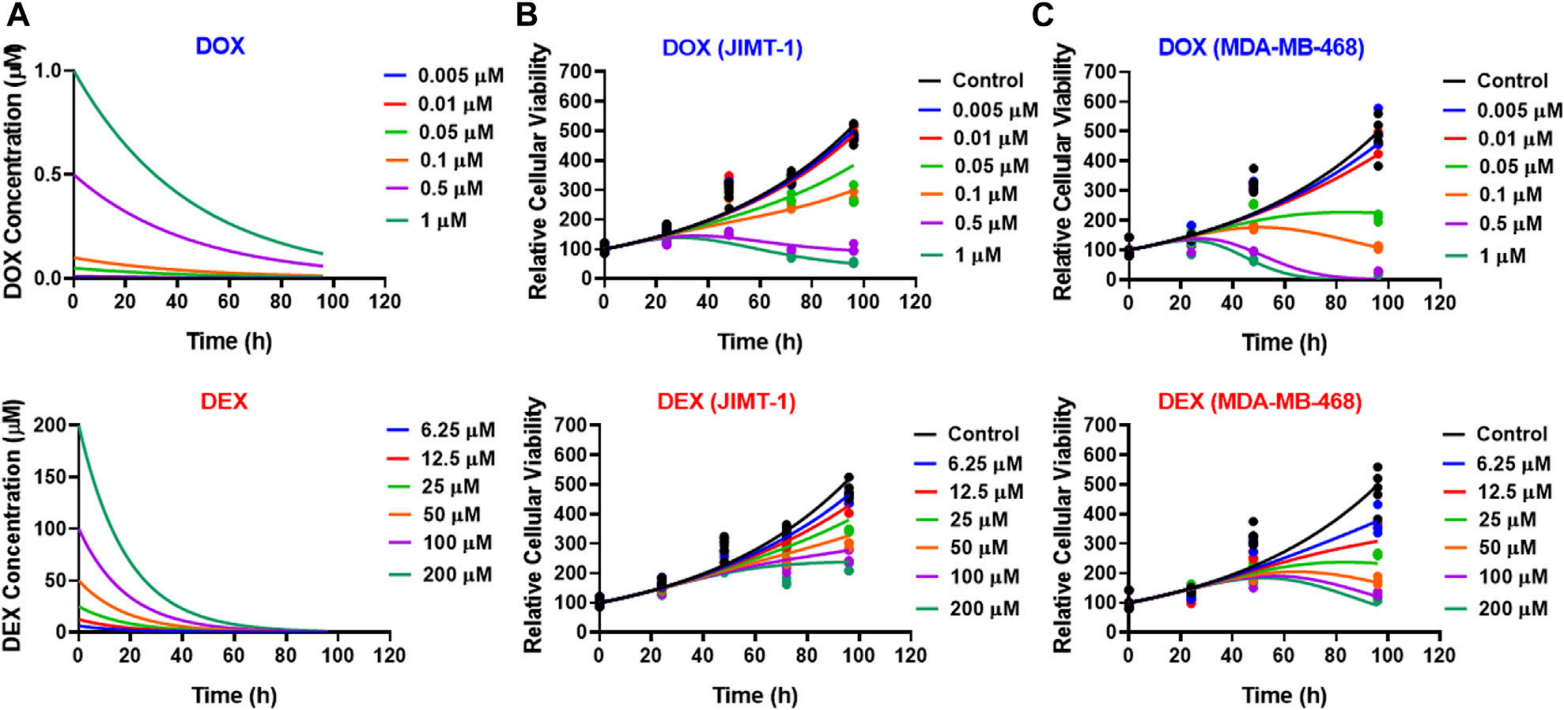
**(A)** Simulated degradation kinetics of DOX (top) and DEX (bottom) at indicated concentrations in a cell culture media. The first-order degradation rate (K_deg_) for DOX and DEX were estimated and assumed to be constant at indicated concentrations. **(B,C)** Model fittings (using PD model) for the *in vitro* effects of the single agents, DOX (top) or DEX (bottom) at indicated concentrations over time on the cell viability of human breast cancer cell lines, JIMT-1 **(B)** and MDA-MB-468 **(C)**. All observed data are represented by solid circles while the smooth lines are model fittings or simulations.

As shown in Figure 3, the cellular level PD response model was developed to simultaneously characterize the time course effects of single agents, DOX or DEX, and their combinations on the cell viability of JIMT-1 and MDA-MB-468. The time-course data (up to 96 h) from six concentrations of DOX (from 0.005 µM to 1 µM), 6 concentrations of DEX (from 6.25 µM to 200 µM) and 36 different DOX and DEX combinations in both the cell lines (consistent with the previous analysis) were simultaneously characterized. The first order, degradation rate constants for DOX (k_deg, DOX_) and DEX (k_deg, DEX_) previously estimated at 0.022 (±0.0004) h^−1^ and 0.054 (±0.0016) h^−1^, respectively, were used to describe their loss in cell culture media over time (Mody et al., 2023). Based on this, the degradation kinetics and the expected change in the concentration profiles of DOX and DEX over time were simulated as shown in Figure 4A. The simulated drug concentration profiles were subsequently used to drive the cellular PD model (Figure 3) to account for loss of drug over time.

**FIGURE 5:**
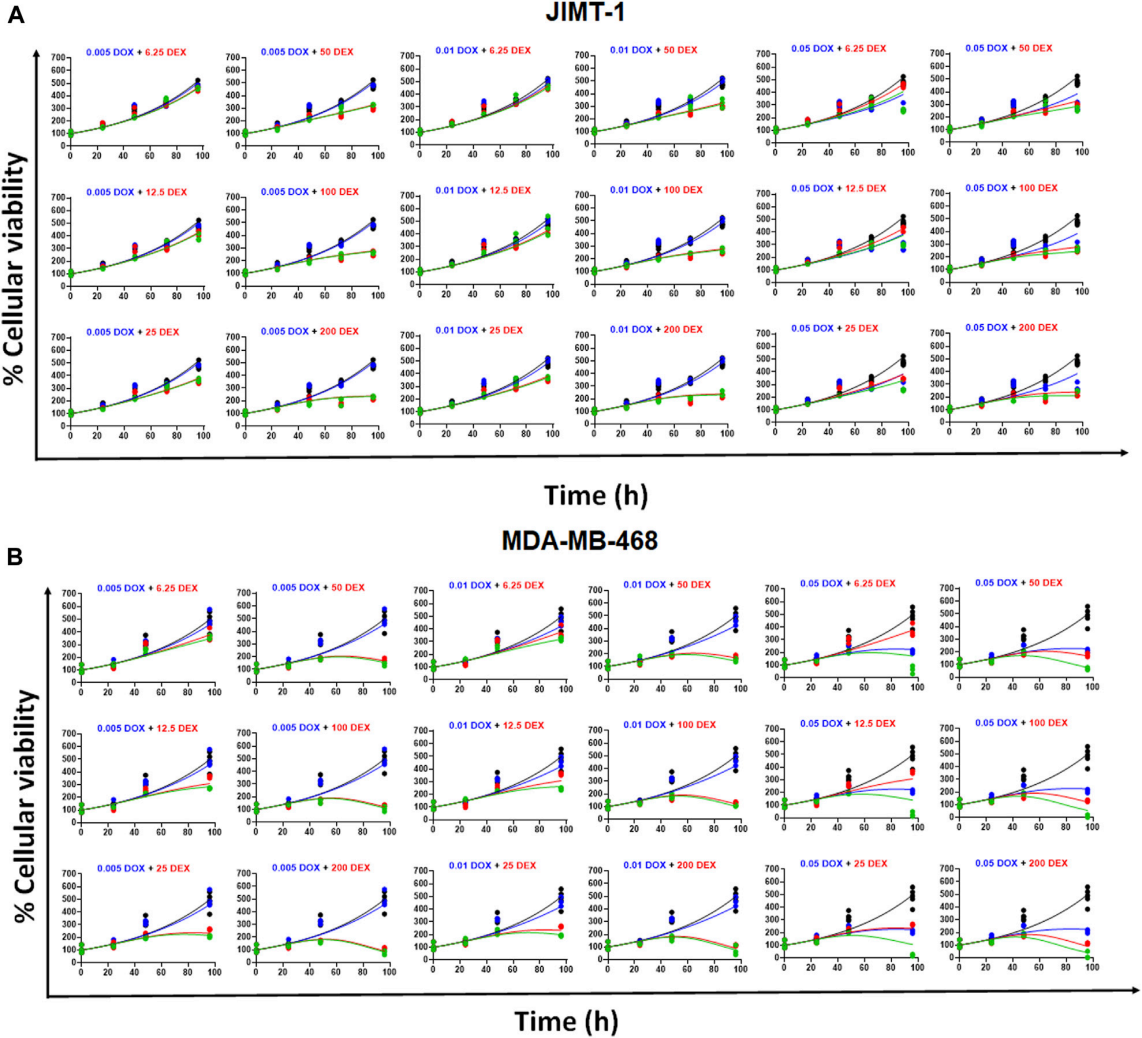
**(A)** Model fittings (using PD model) for the *in vitro* effects of the single agents, DOX, DEX or their combination at indicated concentrations over time on the cell viability of human breast cancer cell line, JIMT-1. All observed data are represented by solid circles while the smooth lines are model fittings or simulations. Black, Control; Blue, DOX; Red, DEX; Green, DOX + DEX. The concentrations used are indicated at the top of each graph for single agents and combinations. **(B)** Model fittings (using PD model) for the *in vitro* effects of the single agents, DOX, DEX or their combination at indicated concentrations over time on the cell viability of human breast cancer cell line, MDA-MB-468. All observed data are represented by solid circles while the smooth lines are model fittings or simulations. Black, Control; Blue, DOX; Red, DEX; Green, DOX + DEX. The concentrations used are indicated at the top of each graph for single agents and combinations.

The model fittings for the time-course effects of DOX or DEX as single agents in JIMT-1 and MDA-MB-468 are shown in Figures 4B, C. The model fittings for the drug combinations (and compared to profiles of single agents) are shown in Figure 5A and Supplementary Figure S2A for JIMT-1 as well as Figure 5B and Supplementary Figure S2B for MDA-MB-468. The model-based parameters were estimated with good precision and are summarized in Table 2. Overall, the model was able to simultaneously capture the data relatively well, as demonstrated by the observations versus individual predictions plot showing roughly uniform distribution of the observed data around the line of identity (Supplementary Figure S3).

**TABLE 2:** Parameter estimates for the *in vitro* cellular-level pharmacodynamic model (PD) for the single and combinatorial effects of Doxorubicin (DOX) and Dexrazoxane (DEX) on human breast cancer cell lines, JIMT-1 and MDA-MB-468.

Parameter (Units)	Definition	Estimate (% RSE)	
		JIMT-1	MDA-MB-468
R_0_ (%)	Baseline % cell viability	100 (Fixed)	100 (Fixed)
k_g_ (hour^−1^)	First-order growth rate constant	0.0171 (1.02)	0.0167 (1.45)
S_max, DOX_ (hour^−1^)	Maximal killing rate constant of DOX	0.0705 (15.6)	1.16 (3.55)
SC_50, DOX_ (µM)	DOX concentration inducing 50% of maximal killing rate	0.315 (18.5)	0.761 (7.48)
1/Ʈ_DOX_ (hour^-1^)	Transit constant for the stimulation of death by DOX	0.0703 (9.9)	0.0275 (3.17)
S_max, DEX_ (hour^−1^)	Maximal killing rate constant of DEX	0.0482 (20.2)	0.29 (48.5)
SC_50, DEX_ (µM)	DEX concentration inducing 50% of maximal killing rate	21.2 (18.8)	17.5 (23.7)
1/Ʈ_DEX_ (hour^−1^)	Transit constant for the stimulation of death by DEX	0.0335 (11.9)	0.0182 (19.4)
ψ	Interaction parameter	∼2 (16.3)	∼1 (0.542)

% RSE, % relative standard error in the model parameters.

In the absence of DOX or DEX, the cell viability was characterized with an exponential growth function (Eq. 3) using a first-order growth rate constant, kg which was estimated to be 0.0171 (±1.02%) h^−1^ and 0.0167 (±1.45%) h^−1^, for JIMT-1 and MDA-MB-468, respectively. The dynamic changes of JIMT-1 and MDA-MB-468 cell viability over 96 h in presence of DOX or DEX were adequately captured with stimulatory effects on cell death characterized with a capacity-limited, Hill function (Eq. 3a-3e). The estimated, maximal killing rate constant (S_max, DOX_) for DOX was 0.0705 (±15.6%) h^−1^ and 1.16 (±3.55%) h^−1^ while the DOX concentration inducing 50% of maximal cell killing rate (SC_50, DOX_) was estimated to be 0.315 (±18.5%) µM and 0.761 (±7.48%) µM for JIMT-1 and MDA-MB-468. On the other hand, the estimated, maximal killing rate constant (S_max, DEX_) for DEX was 0.0482 (±20.2%) h^−1^ and 0.29 (±48.5%) h^−1^ while the DEX concentration inducing 50% of maximal cell killing rate (SC_50, DEX_) was estimated to be 21.2 (±18.8%) µM and 17.5 (±23.7%) µM for JIMT-1 and MDA-MB-468. Thus, time-course data analysis confirmed DOX to be far more potent as compared to DEX which is consistent with the concentration-response analysis. Higher maximal killing rate (S_max_) as well as lower concentration to induce 50% of S_max_ (SC_50_) was consistently estimated for DOX versus DEX in both the cell lines. The delayed effects of DOX and DEX were captured well with the help of three transit compartments on the stimulation of death function, with Ʈ_DOX_ and Ʈ_DEX_ representing the mean transit time. Ʈ_DOX_ was estimated at ∼14 h and ∼ 36 h while Ʈ_DEX_ was estimated at ∼30 h and ∼ 55 h for JIMT-1 and MDA-MB-468.

To characterize the time-course combinatorial effects of DOX + DEX on the cell viability or stimulation of death of breast cancer cell lines, the model structure incorporated an interaction parameter ψ where ψ = 1, ψ < 1, and ψ > 1 indicates additive, synergistic, and antagonistic interactions, respectively (Figure 3; Eq. 4a-4i). The model-based analysis estimated ψ as ∼2 and ∼1 indicating antagonistic and additive interactions for DOX and DEX across different concentration levels tested, in JIMT-1 and MDA-MB-468, respectively. Some minor differences were observed in the estimation of the interaction parameter with the competitive-interaction model-based 3D response surface analysis using static data and cellular-level PD model using time-course data. These could be attributed to the inherent variability arising from the different types of datasets (static versus dynamic) and mathematical approaches used to estimate the interaction parameter. However, it can be concluded that the overall results were largely consistent for the two approaches, suggesting modestly synergistic to additive effects in MDA-MB-468 cells and antagonistic effects in JIMT-1 cells.

### Optimization of DOX and DEX combinations with simulations of clinically relevant dosing regimens

**FIGURE 6:**
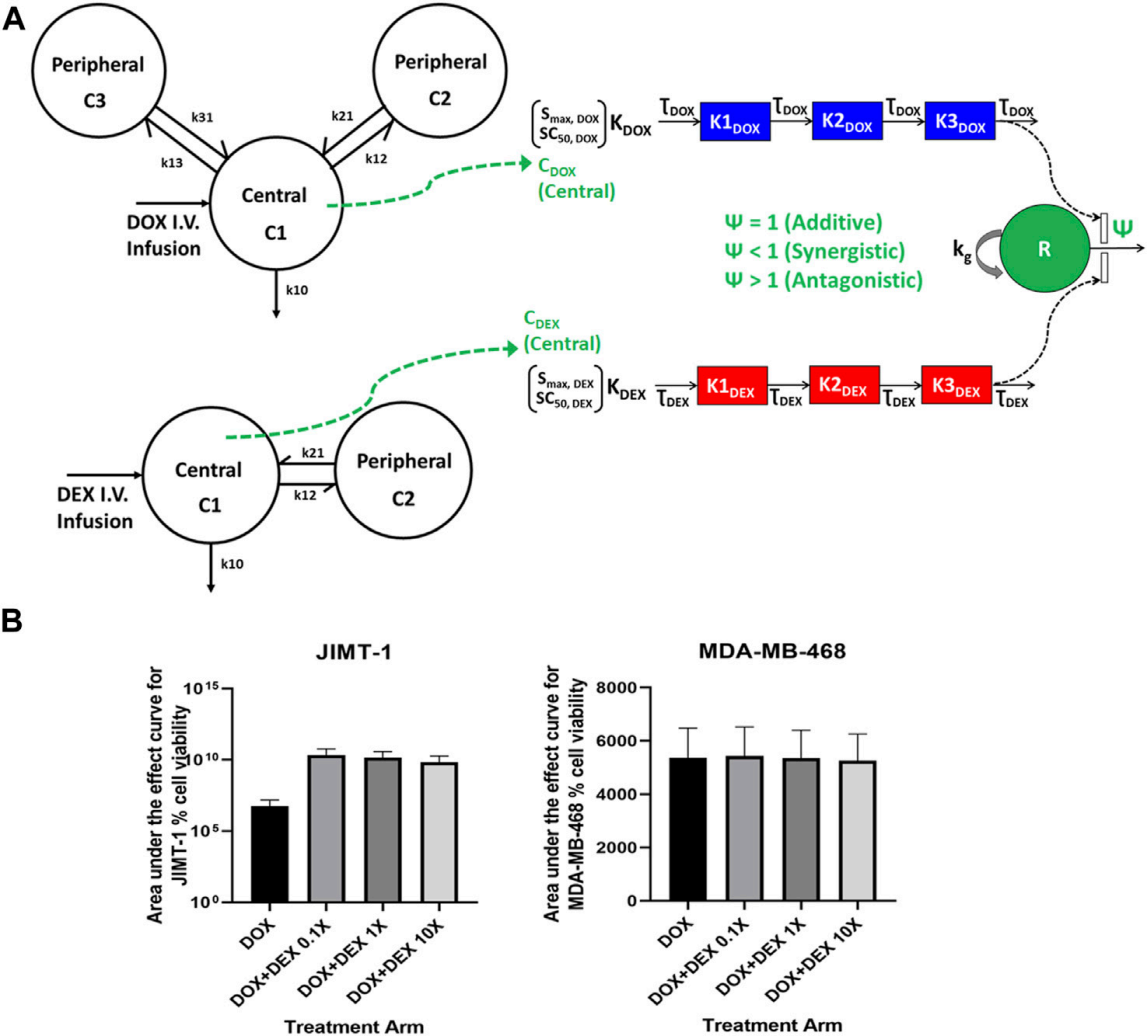
**(A)** Model structure of clinical pharmacokinetics for doxorubicin (DOX) (3-compartment; Left, Top) and for Dexrazoxane (DEX) (2-compartment; Left, Bottom). Schematic of the *in vitro* cellular level pharmacodynamic model (PD) for the single and combinatorial effects of Doxorubicin (DOX) and Dexrazoxane (DEX) (Right) on breast cancer cells. Time-varying concentrations were utilized to drive the DOX + DEX TD model as represented by the green dashed arrow **(B)**. Area under the effect curves for JIMT-1 (left) and MDA-MB-468 (*right*) % cell viability. 50 mg/m^2^ doxorubicin was administered alone or in combination with 500 mg/m^2^ dexrazoxane once every 3 weeks (Q3W) for three dosing cycles. DEX 0.1X, 1X and 10X represent scenarios where tumor concentrations of dexrazoxane were assumed to be 0.1, 1 and 10-fold of plasma concentrations, respectively.

Following establishment of cellular-level PD models for DOX and DEX in JIMT-1 and MDA-MB-468 cells, model-based simulations were performed to investigate the long-term effects of clinically relevant DOX and DEX dosing regimens in tumor cells (Figure 6A). Figure 6B represents area under the effect curves (AUEC) for % cell viability for DOX alone and in combination with DEX for various scenarios of DEX distribution to the tumor site in both cellular models. As described in the *Methods* section, the AUC_tumor_/AUC_plasma_ ratio (Fac_1_) was calculated for DOX based on data extracted from literature (He et al., 2018) and was determined to be 57.1. For DEX, arbitrary ratios (Fac_2_) values of 0.1, 1 and 10 were utilized to describe DEX concentrations at the tumor site. For JIMT-1 cells, the predicted AUEC for % cell viability over three cycles of 3 weeks each was higher with the DOX + DEX combinations (∼1560-fold) as compared to DOX, indicating higher cell killing with DOX alone and antagonistic effects between DOX + DEX on tumor cells, consistent with the *in vitro* PD model (Figure 6B). For MDA-MB-468 cells, the predicted AUEC for % cell viability over three cycles of 3 weeks each was comparable among the DOX and DOX + DEX treatment groups, consistent with the estimated additive effect of DOX + DEX in this cell line *in vitro* (Figure 6B). Of note, the fraction of DEX plasma concentrations distributing to the tumor site (Fac_2_) did not have a significant impact on AUEC predictions of the DOX + DEX combination, likely due to the dominant effect of DOX on tumor cell killing due to its high potency as compared to DEX in tumor cells.

## Discussion

In this manuscript, we presented a proof-of-concept *in vitro* study to examine the nature of drug-drug interaction between DOX and DEX and translated our *in vitro* findings to the clinical setting. Our stepwise approach consisted of first, quantitatively capturing the static concentration-response and time-course anticancer effects of DOX and DEX as single agents. Then, by means of mathematical modeling, we characterized the nature of DOX + DEX interaction by estimating the interaction parameter ψ. We used breast cancer (BC) as a prototype disease and the cell lines JIMT-1 and MDA-MB-468 as prototypes for two difficult-to-treat breast cancer subtypes, BC refractory to HER2 therapy (JIMT-1) and triple negative BC (MDA-MB-468). Second, we performed the *in vitro* to *in vivo* translation of our findings for which we simulated the clinical tumor site concentration-time profiles for DOX and DEX dosing regimens (Earhart et al., 1982; Kontny et al., 2013; He et al., 2018) that were previously identified to offer maximal cardio-protection, and then used the simulated tumor site PK profiles to drive the cell-based PD models. The resulting simulated *in vivo* changes in cancer cell viability (PD responses) were found to be consistent with our *in vitro* findings that a 10:1 DEX:DOX dose ratio over three cycles of Q3W regimen of DOX results in comparable efficacy based on MDA-MB-468 (additive effect) estimates and lower efficacy based on JIMT-1 (antagonistic effect) estimates for DOX + DEX combination as compared to DOX alone. While the developed *in vitro* PD model and *in vivo* translation remains to be validated with future experiments, our previously developed cell-based TD (toxicodynamic) model (Mody et al., 2023) along with the above PD (pharmacodynamic) model can be used to simulate different scenarios and better design future studies to further optimize safe and effective DOX and DEX combinations. Such modeling approaches are extremely useful in more effectively designing *in vivo* studies for different combination regimens (e.g., priming, co-treatment, sequential at different ratios and dose levels) where large preclinical studies are not feasible.

JIMT-1 (HER2 expressing cell line resistant to trastuzumab) and MDA-MB-468 (triple negative breast cancer) were selected as “difficult to treat” breast tumor prototypes and considered representative cell lines for advanced/metastatic breast cancer patients (Tanner et al., 2004; Chavez et al., 2010; Zeichner et al., 2016). In such patients, DOX is typically used at high doses or exposures for the desired antitumor activity and hence higher likelihood of DOX-induced cardiotoxicity. Previous clinical studies have also evaluated the cardioprotective effect of DEX in such advanced breast cancer patients treated with DOX (Marty et al., 2006). While targeted therapies such as trastuzumab are available for HER2 positive tumors, some patients develop resistance which are then treated with chemotherapy such as DOX and hence the use of JIMT-1 as a representative cell line. In addition to DOX, trastuzumab is also known to induce cardiotoxicity, hence previous clinical studies have also explored the cardioprotective use of DEX in patients on DOX and trastuzumab treatment (Kim et al., 2017).

Both the cell lines, JIMT-1 and MDA-MB-468, were previously reported as sensitive to DOX (Wen et al., 2018; Endo et al., 2019). In addition, we identified that DEX alone also induced cytotoxic effects, although at much higher concentrations and with lower maximal killing in the two cell lines (Figure 1). This finding was somewhat consistent with previous studies that reported modest anticancer effects of DEX (Hasinoff et al., 1995; Smith et al., 2016). The estimated *in vitro* IC50 for DOX and DEX in the two cell lines showed ∼500 to 2000 fold higher potency for DOX than DEX (Table 1). Hence, based on these IC50s estimates, a lower range of concentrations for DOX (0.005 µM–1 µM) and a higher range of DEX (6.25 µM–200 µM) were selected for the subsequent single and combination arms studies. These selected concentrations were physiologically relevant (Earhart et al., 1982; Kontny et al., 2013), overlapped with those previously used to evaluate cardiotoxicity in AC16 cardiomyocytes (Mody et al., 2023), and covered a range of efficacies (minimal to maximal) as single agents in the two cancer cell lines. Based on the competitive interaction model, the 3D response surface plots, and the static concentration-response profiles, the interaction between DOX and DEX was estimated to be modestly synergistic in MDA-MB-468 while it was estimated to be modestly antagonistic in JIMT-1 cells (Figure 2).

Next, we developed a cell-level PD model to characterize the time-course effects of DOX and DEX as single agents in the two cancer cell lines. We leveraged previously reported degradation rate constants to account for DOX and DEX loss over time in cell culture media, assuming they are the same across all concentrations. In addition, we assumed that the two drugs do not physically interfere with each other in cell culture media which is consistent with previous reports suggesting that DEX had no impact on the PK and disposition of DOX in rats as well as in breast cancer patients (Rosing et al., 1999; Cusack et al., 2003; Mody et al., 2023). Consistent with static concentration-response studies, the time-course analysis also confirmed superior potency of DOX over DEX as indicated with higher (∼1.5 to 4 folds) maximal killing rate (S_max_) and lower SC_50_ (∼23 to 68 folds) in the two cancer cell lines (Figure 4; Table 2). In addition, MDA-MB-468 was overall observed to be consistently more sensitive to DOX and/or DEX as compared to JIMT-1. As with the competitive interaction model and the 3D response surface analysis, the cell-level PD model also predicted that the interaction of DOX and DEX is additive (ψ = ∼1) and antagonistic (ψ = ∼2) in MDA-MB-468 and JIMT-1, respectively (Figure 5).

The above observations of contrasting DOX and DEX interactions in different cell lines are consistent with few preclinical studies that have previously evaluated the DOX and DEX combination in different cancer types and cell lines. For instance, one study demonstrated that the *in vitro* anti-cancer activity of double strand breaks (DSB) of DOX was mediated by both topoisomerase II alpha (TOP2A) and topoisomerase II beta (TOP2B) isoforms in HTETOP fibrosarcoma cell line (Deng et al., 2014). The same study also confirmed that DEX can negatively impact DOX-induced DSB by depletion of both isoforms. In addition, DEX reduced *in vitro* TOP2A levels and the accumulation of DOX-induced DSB in fibrosarcoma-derived cells but not in lung cancer cells, thereby highlighting the inconsistency of DEX in interfering with the anticancer effects of DOX as was also observed in the current study. The authors argued that the TOP2B depletion may be the primary mechanism by which DEX can potentially interfere with the anticancer activity of DOX. This could be true specifically for breast cancer given the expression of TOP2B in >90% of breast cancer while TOP2A expression is limited in breast tumors as well as since TOP2B rather than TOP2A has been shown to be a better predictor for breast cancer survival in patients (Sandri et al., 1996; Bonnefoi, 2011).

In contrast, another study suggested that DOX and DEX combinations were synergistic in mediating growth inhibition in a HER2 expressing breast cancer cell line, SKBr3. Overall, SKBr3 was more sensitive to DOX than another HER2 expressing breast cancer cell line, BT474; which was consistent with the corresponding levels of TOP2 in those cell lines (Smith et al., 2016). Another report concluded that DEX did not compromise, instead had synergistic interactions with DOX in leukemic cancer cell line, HL-60 (Vavrova et al., 2013). Similarly, the addition of DEX did not impact the cytotoxicity of DOX in MCF-7, another HER2 overexpressing breast cancer cell line (Dallons et al., 2020). Additionally, an *in vivo* study demonstrated that the pre-treatment with DEX (50 mg/kg) did not interfere negatively with the *in vivo* activity of DOX in A2780 and MX-1 human tumor xenograft mouse models (Kurz et al., 2012). These results were largely consistent with another *in vivo* study that concluded DEX did not affect the anticancer activity of DOX in a syngeneic breast tumor rat model (Dickey et al., 2013). Overall, the reduction in tumor volumes and induction of caspase-3 activity were comparable for DOX alone and DOX + DEX combination groups in this study.

Overall, the results related to the impact of DEX on the anti-tumor activity of DOX in the preclinical setting have been variable and still a matter of concern that needs to be addressed. Direct comparison is convoluted by variability in the experimental conditions or design across different studies. For instance, use of one specific DOX and/or DEX concentration or dose levels, specific time points versus kinetic data. In addition, most studies compare DOX alone versus DOX + DEX combination groups to investigate the impact of DEX on the activity of DOX without accounting for the anti-cancer activity contributed by DEX alone. In the present study, we comprehensively investigated the DOX and DEX nature of interactions across multiple concentrations and time points using a combination of experimental data and multiple modeling approaches in breast cancer cell lines.

It should also be noted that DOX and DEX have been shown to induce TOP2 dependent and independent apoptotic effects (Yan et al., 2009). Hence, in addition to TOP2, multiple key protein signaling players may be involved, thereby contributing to the differential interactions of DOX and DEX in different cell lines. As such, the two breast cancer cell lines evaluated in this study have been previously profiled to show differential alterations for genes commonly associated with breast cancer (Sinha et al., 2021) which could be further investigated for their potential role in contributing to the differential and cell line-dependent DOX and DEX interactions. Moreover, the present modeling analysis is a relatively empirical and fit-for-purpose approach towards quantitative characterization of the DOX and DEX interactions in cancer cells wherein cell viability change is used as a surrogate, summing up contributions from different mechanisms (e.g., cell toxicity or cell growth inhibition). Hence, our ongoing mathematical investigations related to the mechanism of action of these drugs (single agent and combination), and thereby their effects on specific cell cycle stages (cytostatic vs. cytotoxic) and relevant protein pathways may help answer the observed discrepancies in sensitivity to DOX and/or DEX in the two cell lines. Further, an enhanced understanding of DOX and DEX molecular mechanisms will not only help optimize and rationalize drug combinations, but also potentially identify a specific patient population where this combination may have wider therapeutic benefits. In addition, leveraging alternative biomarkers as well as additional cell lines representing diverse target cancer patient populations may help to further solidify the findings, and build more confidence while translating findings from *in vitro* to *in vivo* settings and designing subsequent proof-of-concept studies.

Finally, we extended the cell-based PD models to perform clinical simulations and predict the efficacy of long-term dosing regimens on the cell viability of MDA-MB-468 and JIMT-1 cell lines, using the previously identified, optimal cardio-protective dose ratio of DOX and DEX (10:1) (Mody et al., 2023). A clinically relevant DOX dosing regimen of 50 mg/m^2^ administered Q3W alone or in combination with DEX 500 mg/m^2^ over three cycles was chosen for performing model simulations. While clinically relevant tumor-site concentrations of DOX were utilized, for DEX tumor-site concentrations, arbitrary fractions of DEX plasma concentrations were used to drive PD models and perform simulations, due to lack of information regarding tumor tissue to plasma distribution ratios of DEX *in vivo*. However, the fraction of DEX concentrations at the tumor site did not appear to significantly impact cell viability predictions of the DOX + DEX combination, likely due to the dominant effect of DOX on tumor cell killing due to its high potency as compared to DEX. Overall, the tumor cell killing effect of the DOX and DEX combination regimen was demonstrated to be additive in the MDA-MB-468 cell line and antagonistic in the JIMT-1 cell line, reflective of the nature of the interaction observed in the *in vitro* setting, suggesting differential efficacy of this combination *in vivo* depending on the characteristics of tumor cell types and their sensitivity to these agents.

While our *in vitro* to *in vivo* translational modeling approach accounted for clinically relevant DOX and DEX doses and their pharmacokinetic aspects, an important caveat that remains to be addressed is extrapolation of effects at the pharmacodynamic level, given the differences between a static two-dimensional monolayer cell-culture system versus the three-dimensional tumor microenvironment in an *in vivo* system. To this end, next steps and ongoing efforts include extending the *in vitro* assessment of this combination to a three-dimensional and dynamic (3DD) cell culture system, which is more representative of *in vivo* conditions as compared to a standard two-dimensional (2D) monolayer cell culture system (Vaidya et al., 2021). Nevertheless, preliminary exploration of the impact on efficacy *in vivo* using our present *in vitro* PD model as a starting point, provides some insights into the nature of the *in vivo* PD interaction between DOX and DEX in breast cancer cells to guide future studies of this combination.

## Conclusion

To summarize, we have developed a proof-of-concept cell-based PD model which can serve as a platform to optimize DOX and DEX combinations for efficacy indices. As with the TD model, efforts are ongoing to extend the PK model to include different tissue compartments including the tumor and the heart and link appropriate tissue concentrations to PD/TD models so that efficacy and safety predictions can be simultaneously estimated. Similarly, evaluation of the dynamic changes in the intracellular signaling pathway for DOX and DEX PD models is underway to enable better quantitative understanding of the underlying mechanisms, as well as evaluation of this combination in a 3DD system to better reflect *in vivo* conditions. Such developed PK/PD/TD models can serve as *in silico* tools to assess DOX and DEX combinations for safety and efficacy and enable better design of preclinical *in vivo* studies.

## Data Availability

The raw data supporting the conclusion of this article will be made available by the authors, without undue reservation.
