# Supplementary material for: In vitro to clinical translation of combinatorial effects of doxorubicin and dexrazoxane in breast cancer: a mechanism-based pharmacokinetic/pharmacodynamic modeling approach
Source: Front Pharmacol. 2023 Oct 19;14:1239141. doi: 10.3389/fphar.2023.1239141 (PMC10620511; doi:10.3389/fphar.2023.1239141)
Supplement: Supplementary file 1 [file DataSheet1.docx]

**Supplementary Material**

***In Vitro* to Clinical Translation of Combinatorial Effects of Doxorubicin and Dexrazoxane in Breast Cancer: A Mechanism-based Pharmacokinetic/Pharmacodynamic Modeling**

# Approach

Hardik Mody^1†^, Tanaya R. Vaidya^1†^, Jovin Lezeau^1^, Kareem Taha^1^, and Sihem Ait-Oudhia^2^

^†^Contributed equally

^1^Center for Pharmacometrics and Systems Pharmacology, Department of Pharmaceutics, College of Pharmacy, University of Florida, Orlando, Florida, USA.

^2^Quantitative Pharmacology and Pharmacometrics (QP2), Merck & Co., Inc, Rahway, New Jersey, USA.

**Corresponding author:** Sihem Ait-Oudhia, MS. PharmD, PhD. Quantitative Pharmacology and Pharmacometrics (QP2), Merck & Co., Inc, Rahway, New Jersey, USA. Address: 2000 Galloping Hill Rd, Rahway, NJ 07033. Email: sb.manuscript.submission@gmail.com


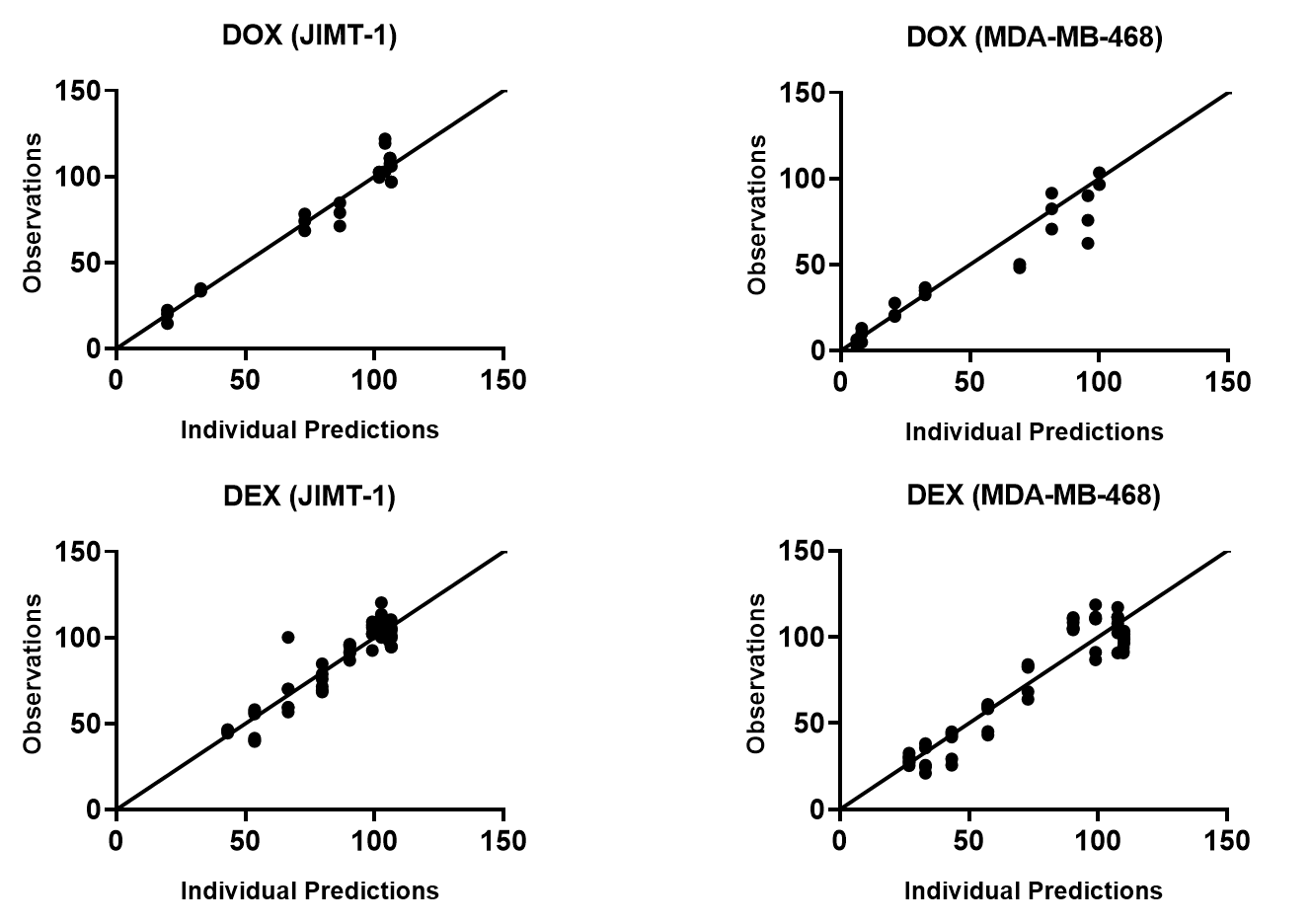


**Supplementary Figure 1.** Observations vs. individual prediction plots for the concentration-response curves for DOX (*top*) and DEX (*bottom*) as single agents in JIMT-1 (*left*) and MDA-MB-468 (*right*) cancer cell lines. The black solid circles represent observed data while the solid line represents the identity line with y=x.


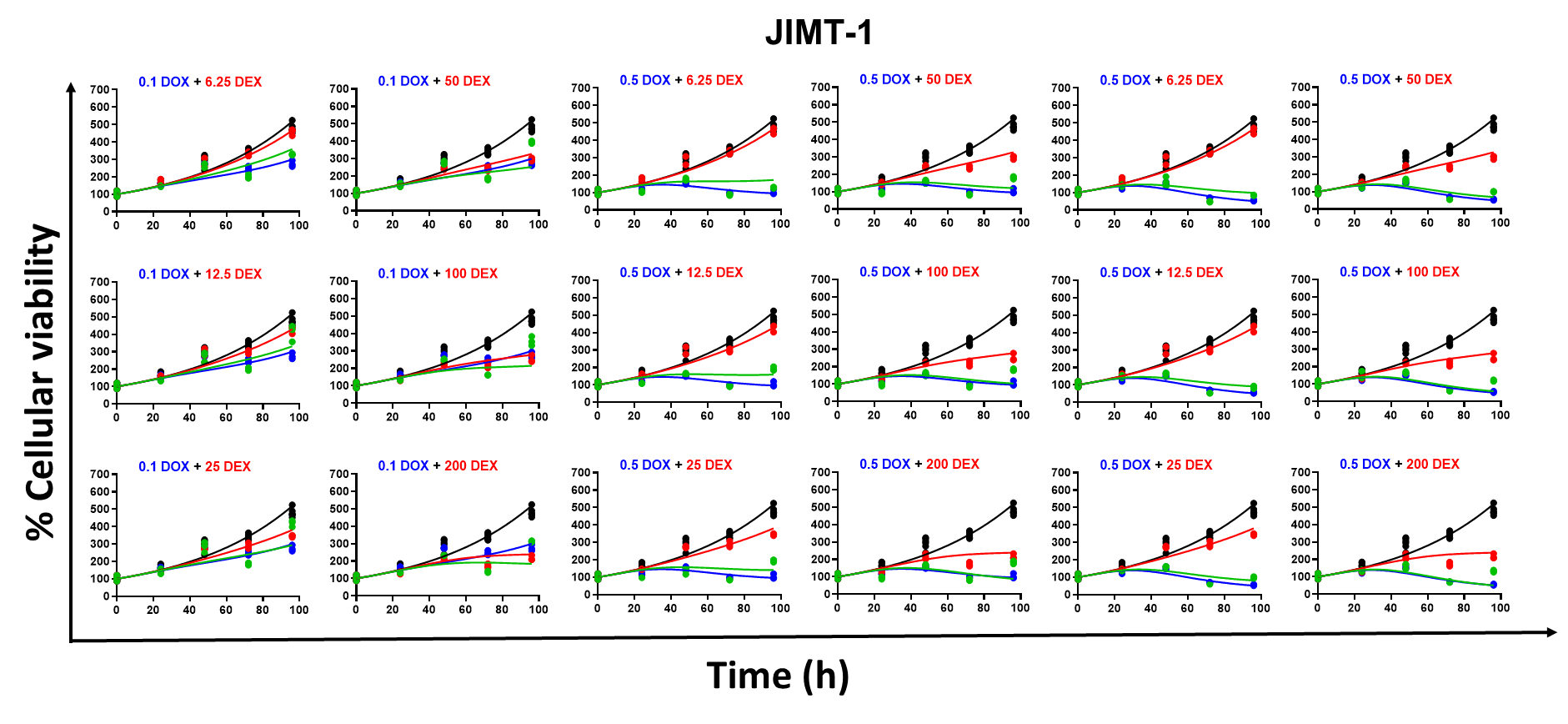


**Supplementary Figure 2. A.** Model fittings (using PD model) for the *in vitro* effects of the single agents, DOX, DEX or their combination at indicated concentrations over time on the cell viability of human breast cancer cell line, JIMT-1. All observed data represented by solid circles while the smooth lines are model fittings or simulations. Black, Control; Blue, DOX; Red, DEX; Green, DOX + DEX. The concentrations used indicated at the top of each graph for single agents and combinations.


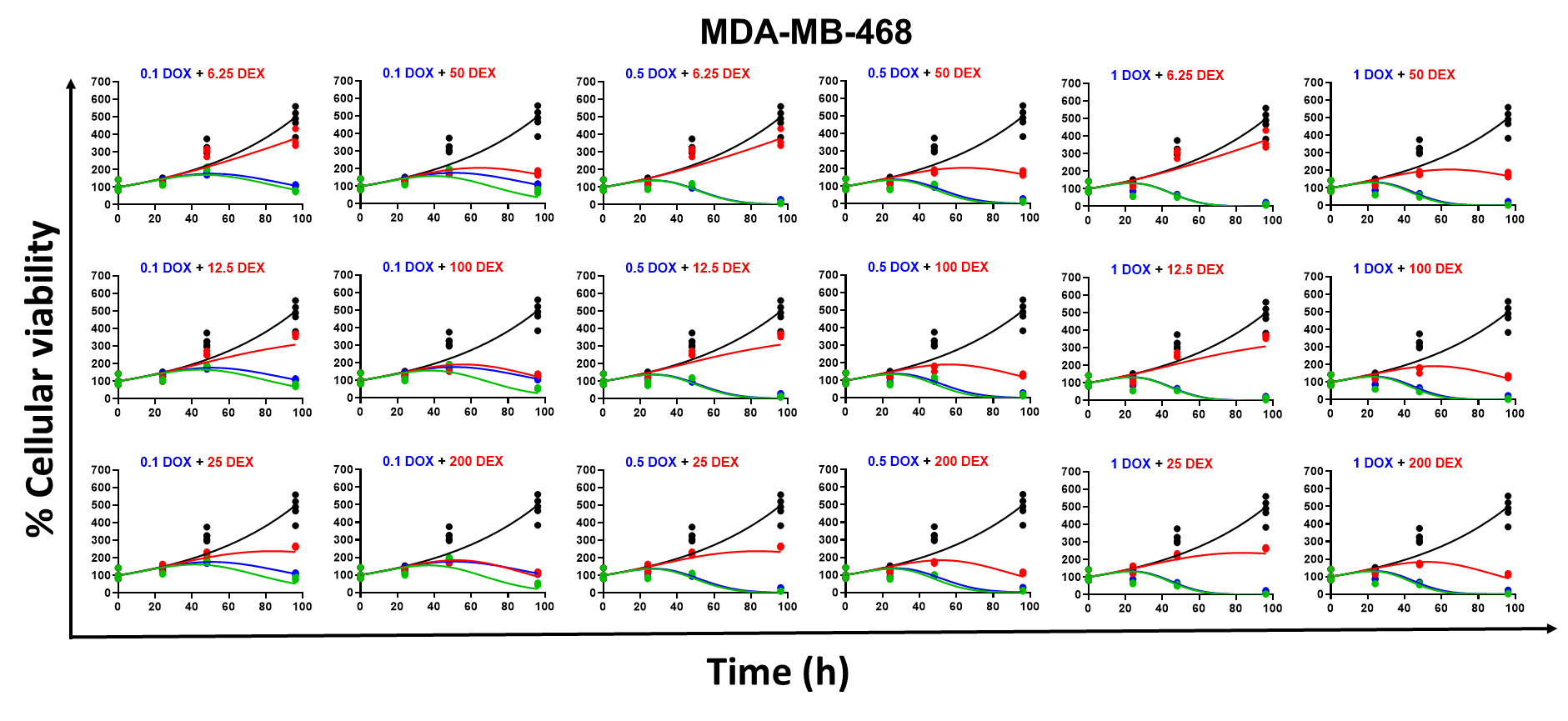


**Supplementary Figure 2. B.** Model fittings (using PD model) for the *in vitro* effects of the single agents, DOX, DEX or their combination at indicated concentrations over time on the cell viability of human breast cancer cell line, MDA-MB-468. All observed data represented by solid circles while the smooth lines are model fittings or simulations. Black, Control; Blue, DOX; Red, DEX; Green, DOX + DEX. The concentrations used indicated at the top of each graph for single agents and combinations.


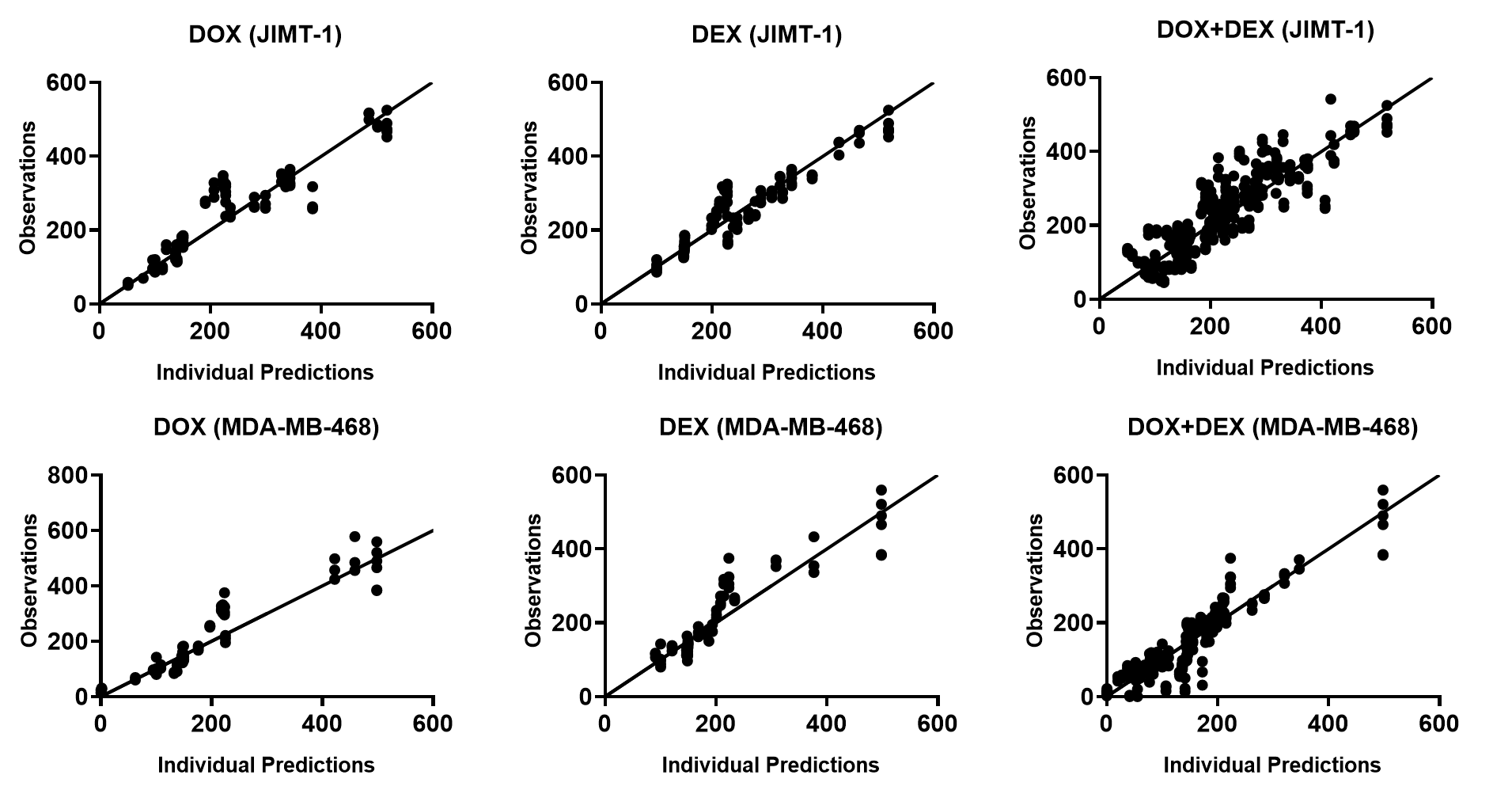


**Supplementary Figure 3.** Observations vs. individual prediction plots for the cell viability (PD model) fittings for DOX (*left*), DEX (*middle*), and DOX+DEX (*right*) in human breast cancer cell lines, JIMT-1 (*top*) and MDA-MB-468 (*bottom*). The black solid circles represent observed data while the solid line represents the identity line with y=x.
